# Predictors of impaired quality of life among colorectal cancer survivors: a cross-sectional study

**DOI:** 10.1186/s41687-025-00971-5

**Published:** 2025-11-25

**Authors:** Abdulrahman Alotaibi, Seon Hahn Kim, Atyaf Alzahrani, Essa Alazmi, Mohammed Musslem, Nada Alharbi, Alaaeldin Darewish, Maha Eltaher, Sakher Abdullah, Shamel Soaida, Tarek Mohammed

**Affiliations:** 1 Department of Surgery, Faculty of Medicine, University of Jeddah, Hamza bin Qasim Street, Al Sharafeyah, P. O. Box: 34, Jeddah, 21959 Saudi Arabia; 2 Department of Surgery, Dr. Soliman Fakeeh Hospital, Jeddah, Saudi Arabia; 3 Department of Surgery, Faculty of Medicine, Universiti Malaya, Kuala Lumpur, Malaysia; 4 Department of Hematology and Oncology, Dr. Soliman Fakeeh Hospital, Jeddah, Saudi Arabia; 5 Department of Radiation Oncology, National Cancer Institute, Cairo University, Cairo, Egypt; 6 Department of Clinical Oncology, Faculty of Medicine, Cairo University, Cairo, Egypt

**Keywords:** Quality of life, Colon cancer, Rectal cancer, Surgery

## Abstract

**Background:**

Comprehensive research on the specific factors affecting the quality of life (QOL) of patients with colorectal cancer (CRC) is lacking. We investigated patient factors affecting QOL in this population.

**Methodology:**

We conducted a cross-sectional study at Dr. Suliman Fakeeh Hospital, a private tertiary center in Jeddah City, KSA. We used the validated Arabic versions of the Cancer Quality of Life Questionnaire-30 (QLQ-30) and Colorectal Cancer Quality of Life Questionnaire-29 (QLQ-CR29) in patients with CRC. We used a generalized linear model to analyze the data. Significant linear correlations between functional and symptom scores and 11 factors, including age, sex, tumor site and stage, comorbidities, surgery type/approach, leaks, stoma, and neoadjuvant or adjuvant therapy, were detected.

**Results:**

A total of 104 participants answered the survey. The mean global health score was 72.43 (95% confidence interval [CI] 68.18–76.52). Cognitive and physical function scored highest and lowest on the QLQ-30, with a mean of 76.92 (95% CI 71.47–81.73) and 24.92 (95% CI 65.57–74.61), respectively. The worst symptom was insomnia, with a mean of 42.30 (95% CI 36.21–48.71). The most distressing symptoms on the QLQ-CR29 were stoma embarrassment and bloating, with a mean of 41.34 (95% CI 24.67–58.01). Tumor stage and stoma were the two most significant factors for predicting poor QOL. The factors influencing impotence and male libido included age, tumor stage, comorbidities, and the use of chemoradiotherapy.

**Conclusions:**

A one-unit increase in tumor stage was correlated with a 4.88-unit decrease in the global health status score, whereas a stoma was linked to a 22-fold reduction in social function scores. Advanced tumor stage and the presence of a stoma significantly affected patients’ functional status and symptoms.

**Supplementary Information:**

The online version contains supplementary material available at 10.1186/s41687-025-00971-5.

## Background

The number of colorectal cancer (CRC) cases among Saudi nationals in 2020 was 1729, with 35% (613/1729) of these cases being rectal cancer [1], affecting 966 (55.9%) men and 763 (44.1%) women. Any individual who overcomes cancer is considered a survivor according to the National Cancer Institute [2]. Macmillan Cancer Support (UK) defines a cancer survivor as a person who is “living with or beyond cancer,” including individuals who have completed cancer treatment with no active disease, those currently undergoing treatment but not at the terminal stage, and those with a history of cancer [3]. Overall, 1.7 million individuals in the United States have survived CRC as of January 2022, with two-thirds of them being aged ≥ 65 years [4].

CRC treatment has physical and psychological repercussions, such as gastrointestinal complications, incontinence, sexual dysfunction, discomfort, exhaustion, emotional distress, and challenges related to insurance, employment, and disability. Therefore, quality of life (QOL) evaluations in patients with cancer have garnered interest in clinical practice owing to their ability to provide insights into the impact of cancer and its treatments on patients’ overall health and well-being [2, 4, 5]. QOL assessments aid in customizing treatment plans to address the unique needs of patients. The long-term effects of cancer diagnosis and treatment on mental health, physical well-being, social support, and functional ability contribute to lower QOL [5, 6].

Only a few national studies have used the QLQ-CR30 and CR29 questionnaires to assess QOL among CRC survivors [7–11]. Since these studies were performed shortly after treatment completion, they reported borderline global QOL. Some studies lacked a comprehensive representation, as they tended to include more people with colon tumors or early-stage cancer than those with advanced or metastatic cancer [9, 10], whereas other studies did not provide information regarding tumor stages [7, 8, 11]. Therefore, long-term QOL after one year of treatment completion remains unknown and there is a need to fill this literature gap.

The aim of this study was to investigate the effects of patient factors, such as age, sex, comorbidities, tumor site and stage, surgery type, postoperative leak complications, stoma presence, and chemoradiotherapy, on the QOL of patients with CRC using the Cancer Quality of Life Questionnaire-30 (QLQ-30) and Colorectal Cancer Quality of Life Questionnaire-29 (QLQ-CR29).

## Methods

### Study design and patients

Patients with CRC treated at Dr. Suliman Fakeeh Hospital, a private tertiary center in Jeddah, were included in this cross-sectional study. We collected patient contact data between October 2018 and October 2022. The inclusion criteria were patients aged > 18 years; diagnosed with CRC via histopathological biopsy and imaging (stages I–IV); currently undergoing treatment for curative or semicurative CRC or have completed full treatment and follow-up at our centers; with adequate cognitive functions capable of articulating their condition; completed one year since the initiation of treatment; and provided consent for participation. Patients receiving palliative care, those with less than one year of follow-up, individuals unable to comprehend questions owing to cognitive impairments, and patients with incomplete data were excluded. In total, 253 patients were identified at the start of data collection. We excluded 47 patients owing to their deceased status. Moreover, 70 patients who were either unreachable or did not complete the hospital’s follow-up process were excluded. The follow-up process requires the patient to visit the clinic every three months for the first 2 years post treatment, followed by visits every 4–6 months until 5 years post treatment.

Overall, 136 patients from a single private center were invited to participate in the study. The target sample size of 101 participants had a margin of error of 5% for 136 patients with CRC and a confidence level of 95%.

### Data collection and questionnaires

We selected standardized surveys owing to their widespread use and established dependability in cancer research. These instruments accurately and reliably measure various dimensions of QOL. The European Organization for the Research and Treatment of Cancer (EORTC) developed validated Arabic versions of the QLQ-30 (Cancer Quality of Life Questionnaire) and QLQ-CR29 (Colorectal Cancer Quality of Life Questionnaire) (https://qol.eortc.org/questionnaires/) [12, 13]. Trained researchers, specifically physicians, conducted interviews with the study participants and requested them to complete the survey.

The hospital’s electronic system provided additional data on each patient. The records included information regarding the patient’s tumor site, stage, comorbidities, surgery type and approach, postoperative complications, stoma diversion, and the need for neoadjuvant or adjuvant therapy.

### Statistical analysis

We calculated the means, standard deviations, and 95% confidence intervals (CIs) for each component of the questionnaires. We depicted the frequency and proportion of respondents falling into the categories of poor and excellent scores.

Considering the continuous nature of the scores, we used a generalized linear model to investigate significant linear correlations between functional and symptom scores and 11 other factors, including age, sex, tumor site and stage, comorbidities, surgery type and approach, leak as a complication, stoma or diversion, and the administration of neoadjuvant and adjuvant therapy. Statistical significance was set at *P* < .05. We used IBM SPSS version 25 for data analysis. Positive significant regression coefficients had a greater effect on reference values than negative ones did. The values used as references included male sex, colon tumor status, stage 0 disease, no comorbidities, no complications, no surgery, open surgery, no stoma, and no adjuvant or neoadjuvant therapy.

## Results

### Demographic data of the study group

Overall, 104 patients participated. The mean duration of follow-up was 38 (12–60) months. The mean age of the participants was 57.3 (27–86) years. The proportion of young patients (< 50 years) was 28.8% (30/104). Males comprised 72.1% of the patients (75/104). Moreover, 42.3% (44/104) of the tumors were in the rectum, whereas 57.7% (60/104) were in the colon. Young participants had a rectal-to-colon tumor ratio of 1:1, whereas the overall sample ratio was 1:1.3.

In terms of TNM tumor stage, stages I, II, III, and IV were present in 9.6%, 26.9%, 46.2%, and 17.3% of patients, respectively. Moreover, 39.4% (41/104) received neoadjuvant therapy, whereas 40.4% (42/104) required adjuvant therapy.

The most frequently performed surgical procedures were anterior resection (AR) and low anterior resection (LAR) for distal sigmoid and upper rectal tumors (30.8%), followed by right hemicolectomy (RHC) for cecal and ascending colon tumors (27.9%) and ultralow anterior resection (uLAR) for mid- and lower rectal cancer (18.3%). Among all the participants, 11.5% (12/104) did not receive surgery owing to a complete response to neoadjuvant therapy, a high risk for surgery, or the choice not to undergo surgery. Notably, 59.7% (55/92) of patients who underwent surgery selected minimally invasive techniques.

Overall, 5.4% (5/92) of the patients had postoperative complications, such as leaks. Moreover, 39.4% (41/104) of the patients received temporary stoma diversion, and four patients required permanent stoma placement. Adjuvant chemotherapy was administered to 40.4% (42/104) of the participants. The patients’ demographic data are summarized in Table 1.

**Table 1:** Demographic data and global quality of life score of participants (*N* = 104)

Characteristics	Number (% or range)	Global quality of life					*P* value*
		Poor (< 33%)		Average (33–66%)	Excellent (> 66%)		
Age (mean)	57.4 (27–86)						
Age							0.259
Less than 50 y (Young)	30 (28.8%)	2 (6.7%)	18 (60%)			10 (33.3%)	
More than 50 y	74 (71.2%)	3 (4.1%)	56 (75.6%)			15 (20.3%)	
Sex							0.447
Male	75 (72.1%)	5 (6.7%)	53 (70.6%)			17 (22.7%)	
Female	29 (27.9%)	0 (0%)	21 (72.4%)			8 (27.6%)	
Tumor Site							0.185
Colon	60 (57.7%)	2 (3.3%)	47 (78.4%)			11 (18.3%)	
Rectum	44 (42.3%)	3 (6.8%)	27 (61.4%)			14 (31.8%)	
Comorbidities							0.323
No	65 (62.5%)	2 (3.1%)	45 (69.2%)			18 (27.7%)	
Yes	39 (37.5%)	3 (7.7%)	29 (74.4%)			7 (17.9%)	
Tumor stage (TNM)**							0.286
I	10 (9.6%)	0 (0%)	10 (100%)			0 (0%)	
II	28 (26.9%)	2 (7.1%)	20 (71.5%)			6 (21.4%)	
III	48 (46.2%)	2 (4.2%)	34 (70.8%)			12 (25%)	
IV	18 (17.3%)	1 (5.6%)	10 (55.5%)			7 (38.9%)	
Surgery type***							0.489
RHC	29 (27.9%)	2 (6.9%)	23 (79.3%)			4 (13.8%)	
LHC	5 (4.8%)	0 (0%)	4 (80%)			1 (20%)	
AR/LAR	32 (30.8%)	0 (0%)	25 (78.1%)			7 (21.9%)	
uLAR	19 (18.3%)	2 (10.5%)	10 (52.7%)			7 (36.8%)	
APR	4 (3.8%)	0 (0%)	3 (75%)			1 (25%)	
TAC/TPC	3 (2.9%)	0 (0%)	2 (66.7%)			1 (33.3%)	
No surgery	12 (11.5%)	1 (8.3%)	7 (58.3%)			4 (33.3%)	
Leak (complication)							0.128
No	87 (94.6%)	4 (4.6%)	65 (74.7%)			18 (20.7%)	
Yes	5 (5.4%)	0 (0%)	2 (40%)			3 (60%)	
Approach							0.356
No surgery	12 (11.5%)	1 (8.3%)	7 (58.3%)			4 (33.3%)	
Laparoscopic	55 (52.9%)	1 (1.8%)	42 (76.4%)			12 (21.8%)	
Open	37 (35.6%)	3 (8.1%)	25 (67.6%)			9 (24.3%)	
Stoma (diversion)							0.171
No	63 (60.6%)	2 (3.2%)	49 (77.8%)			12 (19%)	
Yes	41 (39.4%)	3 (7.3%)	25 (61%)			13 (31.7%)	
Neoadjuvant therapy							0.128
No	63 (60.6%)	3 (4.8%)	49 (77.8%)			11 (17.6%)	
Yes	41 (39.4%)	2 (4.9%)	25 (61%)			14 (34.1%)	
Adjuvant therapy							0.550
No	60 (57.7%)	3 (5%)	45 (75%)			12 (20%)	
Yes	42 (40.4%)	2 (4.8%)	28 (66.7%)			12 (28.7%)	

* Chi-squared or Fisher’s exact test

** TNM: Tumor, lymph node, and metastasis staging system, which was developed and is maintained by the Union for International Cancer Control (UICC)

***Surgery type; RHC; right hemicolectomy, LHC; left hemicolectomy, AR; anterior resection LAR; low anterior resection, uLAR; ultralow anterior resection (for mid and low rectal cancer), APR; abdominoperineal resection, TAC; total abdominal colectomy, TPC; total proctocolectomy

### QLQ-30

The global health status mean score (standard deviation, SD) was 72.43 (22.15), with a 95% CI of 68.18–76.52. Cognitive function exhibited the highest functional scores, followed by role, emotional, and social functions. The mean (95% CI) values for these scores were 76.92 (95% CI 71.47–881.73), 75.32 (95% CI 70.19–880.44), 69.15 (95% CI 63.22–774.99), and 63.78 (95% CI 57.05–770.19), respectively. Physical function had the lowest functional score, with a mean of 58.53 (95% CI 65.57–74.61).

In terms of symptom scores, insomnia had the highest average score, followed by fatigue, constipation, and pain. The mean (95% CI) values for these symptoms were 42.30 (95% CI 36.21–448.71), 38.88 (95% CI 33.01–444.76), 36.12 (95% CI 29.48–443.91), and 33.01 (95% CI 27.56–338.77), respectively. The data obtained from this questionnaire are summarized in Table 2.

**Table 2:** Mean score of all items in the QLQ-C30 and QLQ-CR29 (*N*=104)

Variables	*N*	Mean (SD)	95% CI	*N* (%) scoring < 33.3^a^	*N* (%) scoring ≥ 66.6^a^
QLQ-C30*					
Global health status/QoL	104	72.43 (22.15)	68.18–76.52	3 (2.9)	74 (71.2)
A) Functional scales^b^					
Physical function	104	58.53 (24.92)	65.57–74.61	13 (12.5)	60 (57.6.)
Role function	104	75.32 (28.15)	70.19–80.44	4 (3.8)	79 (76.0)
Emotional function	104	69.15 (30.16)	63.22–74.99	11 (10.6)	71 (68.3)
Cognitive function	104	76.92 (27.11)	71.47–81.73	6 (5.8)	84 (80.8)
Social function	104	63.78 (34.40)	57.05–70.19	15 (14.4)	66 (63.5)
B) Symptoms scales^c^					
Fatigue	104	38.88 (30.30)	33.01–44.76	42 (40.4)	24 (23.1)
Nausea and vomiting	104	15.86 (25.26)	11.05–21.15	80 (76.9)	8 (7.7)
Pain	104	33.01 (29.55)	27.56–38.77	49 (47.1)	20 (19.2)
Dyspnea	104	19.55 (30.67)	13.78–25.95	66 (63.5)	15 (14.4)
Insomnia	104	42.30 (33.24)	36.21–48.71	24 (23.1)	35 (33.7)
Loss of appetite	104	25.00 (32.43)	19.23–31.41	55 (52.9)	19 (18.3)
Constipation	104	36.21 (39.44)	29.48–43.91	46 (44.2)	33 (31.7)
Diarrhea	104	28.20 (32.10)	22.11–34.61	48 (46.2)	23 (22.1)
Financial difficulties	104	27.88 (33.53)	21.79–34.92	52 (50.0)	25 (24.0)
QLQ-CR29**					
A) Functional scales^b^					
Body image	104	70.62 (30.22)	64.74–75.85	12 (11.5)	73 (70.2)
Anxiety	104	95.03 (4.05)	92.23–95.80	0	93 (89.4)
Weight	104	60.57 (38.23)	53.84–67.62	21 (20.2)	67 (64.4)
Male sex interest	104	68.27 (47.68)	58.65–77.24	14 (13.5)	56 (53.8)
B) Symptoms scales^c^					
Urinary frequency	104	2.26 (0.85)	2.10–2.43	6 (5.7)	85 (81.7)
Blood and mucus in stool	104	13.78 (27.72)	8.97–19.23	78 (75.0)	11 (10.6)
Urinary incontinence	104	14.74 (28.94)	9.30–20.19	77 (74.0)	12 (11.5)
Dysuria	104	15.06 (26.22)	10.25–20.19	73 (70.2)	13 (12.5)
Abdomen pain	104	33.33 (34.13)	27.24–40.05	42 (40.4)	30 (28.8)
Buttock pain	104	26.92 (33.52)	20.83–33.97	55 (52.9)	26 (25.0)
Bloating	104	41.34 (34.91)	34.62–48.06	31 (29.8)	40 (38.5)
Dry mouth	104	23.07 (30.21)	26.02–33.45	91 (87.5)	0
Hair loss	104	21.15 (32.2)	14.75–27.55	66 (63.5)	20 (19.2)
Taste	104	13.78 (25.27)	9.29–18.90	74 (71.2)	9 (8.7)
Flatulence	104	18.65 (56.89)	10.26–32.19	82 (78.8)	15 (14.4)
Fecal incontinence	104	36.53 (90.12)	19.55–53.84	69 (66.3)	25 (24.0)
Sore skin	104	35.89 (86.85)	19.23–53.51	84 (80.8)	12 (11.5)
Embarrassment	104	41.34 (86.71)	24.67–58.01	82 (78.8)	16 (15.4)
Impotence	104	21.79 (46.16)	12.50–30.12	54 (51.9)	30 (28.8)
Dyspareunia	104	40.51 (28.37)	25.63–64.74	94 (90.4)	6 (5.8)

* QLQ-C30: Quality of Life Questionnaire Cancer (30-item)

** QLQ-CR29: Quality of Life Questionnaire Colorectal (29-item)

^a^: Regarding functional scales, a score of < 33.3% exhibited problems, while ≥ 66.7% demonstrated good functioning. Regarding symptom scales, participants who scored < 33.3% demonstrate good functioning, while those who scored ≥ 66.7% exhibit problems

b: Good functioning is indicated by the highest mean score in functional scales

c: Poor symptoms are indicated by the highest mean score in symptom scales

### QLQ-CR29

According to the CRC functional scale, anxiety had the highest score and best functioning, with a mean of 95.03 (95% CI 92.23–95.80). Weight loss was the most concerning issue, with a mean score of 60.57 (95% CI 53.84–667.62). The mean scores for male libido and body image issues exceeded 66.6, with scores of 68.27 (95% CI 58.65–77.24) and 70.62 (95% CI 64.74–75.85), respectively. In terms of symptoms, the symptoms causing the most distress were stoma embarrassment and bloating, with a mean score of 41.34 (95% CI 24.67–58.01), followed by dyspareunia, with a mean score of 40.51 (95% CI 25.63–664.74). Patients demonstrated satisfactory outcomes regarding hair loss, impotence, and urinary and fecal incontinence. The data obtained from this questionnaire are summarized in Table 2.

### Predictive parameters of the QLQ-30 and QLQ-CR29

We investigated the correlations of 11 parameters with the QLQ-30 and QLQ-CR29 scores using linear regression, as follows:

#### Age

This cohort had a mean age of 57 years. Age significantly affected only men’s libido. A 0.8-fold reduction in libido was observed for every 1-year increase in age (*P* = .001).

#### Sex

Women scored higher for physical, emotional, and cognitive functioning (*P* = .029, 0.003, and 0.037, respectively). More women reported nausea/vomiting and bloating (*P* = .031 and 0.015, respectively). Compared with men, women had a 12-fold greater likelihood of experiencing pain (*P* = .04) and a 24-fold greater likelihood of reporting hair loss (*P* = .001).

#### Tumor site

Rectal tumors significantly increased buttock pain and skin sores by 22- and 40-fold (*P* = .002 and 0.044), respectively.

#### Tumor stage

Tumor stage primarily affected global health status. A 4.88-unit decline in the global health status score was observed for every one-unit increase in disease stage (*P* = .038). Advanced stages had a significant negative impact on physical, role, emotional, and social functions; patient weight; and impotence (*P* = .002, 0.03, 0.036, 0.005, 0.002, and 0.035, respectively). Stage had a significant impact on fatigue, nausea and vomiting, pain, insomnia, loss of appetite, financial difficulties, body image issues, male libido, dysuria, hair loss, and taste.

#### Comorbidities

Comorbidity was associated with a 12-fold greater reduction in physical function than in healthy patients (*P* = .018). Comorbidity negatively affected impotence 19-fold compared with no comorbidity (*P* = .004).

#### Leak

The occurrence of a leak did not significantly affect patient QOL.

#### Approach

The surgical approach had a significant effect on emotional functioning, dyspnea, constipation, weight loss, urinary frequency, fecal incontinence, and skin sores (*P* = .023, 0.048, 0.002, 0.047, 0.015, 0.017, and 0.022, respectively).

#### Stoma

Stomas reduced role functioning by 20 units and emotional functioning by 18 units (*P* = .003 and 0.014, respectively). Social functioning decreased by 22 units (*P* = .007). A stoma was strongly associated with dyspnea, reduced appetite, bloating, dry mouth, and skin soreness. These associations were strong (*P* = .045, 0.041, 0.027, 0.042, and 0.002, respectively). Stomas did not affect patients’ physical function or financial difficulties.

#### Radiation and chemotherapy

The emotional functioning of patients who received neoadjuvant therapy significantly decreased by 26 units (*P* = .002). Chemoradiotherapy treatment was associated with abdominal pain and impotence (*P* = .017 and 0.002, respectively). Tables 3 and 4 describe the predictive parameters for the QLQ-30 and QLQ-CR29, respectively.

**Table 3:** Predictors in QLQ-C30 (*N*=104)

**Parameter estimates**	**Global health status/QOL**		**Physical function**			**Role function**		**Emotional function**		**Cognitive function**	
	**B (95% CI)**	***P*** ** value**	**B (95% CI)**		***P*** ** value**	**B (95% CI)**	***P*** ** value**	**B (95% CI)**	***P*** ** value**	**B (95% CI)**	***P*** ** value**
Age	0.171	0.359	-0.21		0.301	0.316	0.17	0.153	0.535	0.11	0.642
Sex	-0.626	0.894	11.119		**0.029**	10.129	0.08	18.188	**0.003**	12.385	**0.037**
Tumor site	-0.572	0.911	2.809		0.614	-5.024	0.427	-9.774	0.148	-0.974	0.881
Stage	-4.887	**0.038**	-7.79		**0.002**	-6.325	**0.03**	-6.528	**0.036**	-4.685	0.117
Comorbidities	-7.144	0.131	-12.182		**0.018**	-11.122	0.057	-3.948	0.528	-5.932	0.324
Type of surgery	-1.111	0.106	-0.387		0.605	-0.89	0.295	-0.479	0.598	0.32	0.714
Leak (complication)	-5.005	0.218	-1.723		0.697	4.784	0.341	-3.844	0.474	-3.602	0.485
Approach	-0.604	0.779	0.984		0.674	-0.154	0.954	6.459	**0.023**	4.771	0.08
Stoma (diversion)	-5.916	0.284	-7.611		0.205	-20.171	**0.003**	-17.993	**0.014**	-9.867	0.16
Neoadjuvant therapy	0.134	0.983	8.098		0.243	14.083	0.074	-26.329	**0.002**	11.944	0.141
Adjuvant therapy	-0.035	0.994	7.44		0.139	4.808	0.4	1.283	0.834	-2.417	0.681
**Parameter estimates**	**Social function**		**Fatigue**			**Nausea and vomiting**		**Pain**		**Dyspnea**	
	**B (95% CI)**	***P*** **value**	**B (95% CI)**	***P*** **value**		**B (95% CI)**	***P*** **value**	**B (95% CI)**	***P*** **value**	**B (95% CI)**	***P*** **value**
Age	0.21	0.458	-0.124	0.635		0.026	0.904	0.028	0.912	0.029	0.913
Sex	6.992	0.324	-6.872	0.293		-11.51	**0.031**	-12.942	**0.04**	-9.759	0.139
Tumor site	-4.289	0.581	1.797	0.802		-4.289	0.462	6.16	0.372	-2.353	0.744
Stage	-10.017	**0.005**	8.479	**0.01**		7.341	**0.006**	7.183	**0.024**	5.449	0.101
Comorbidities	-4.893	0.495	6.167	0.351		-2.829	0.6	4.772	0.455	8.389	0.209
Type of surgery	-0.951	0.362	0.624	0.516		1.07	0.173	1.255	0.176	0.626	0.518
Leak (complication)	-7.868	0.202	3.924	0.49		4.327	0.351	4.664	0.395	5.474	0.339
Approach	0.166	0.959	-2.001	0.505		-4.156	0.09	-1.707	0.556	-5.997	**0.048**
Stoma (diversion)	-22.485	**0.007**	13.831	0.073		5.794	0.358	8.145	0.275	15.637	**0.045**
Neoadjuvant therapy	17.285	0.074	-11.893	0.182		-14.085	0.053	-15.13	0.079	-11.292	0.209
Adjuvant therapy	5.781	0.41	-3.941	0.542		-9.968	0.059	-4.386	0.482	3.486	0.593
**Parameter estimates**	**Insomnia**		**Loss of appetite**			**Constipation**		**Diarrhea**		**Financial difficulties**	
	**B (95% CI)**	***P*** **value**	**B (95% CI)**	***P*** **value**		**B (95% CI)**	***P*** **value**	**B (95% CI)**	***P*** **value**	**B (95% CI)**	***P*** **value**
Age	0.418	0.146	0.262	0.342		0.323	0.336	-0.218	0.437	-0.305	0.273
Sex	-13.24	0.066	-3.858	0.577		-9.25	0.271	-4.204	0.549	4.644	0.505
Tumor site	-5.701	0.469	-10.778	0.154		15.786	0.086	11.009	0.151	10.893	0.152
Stage	7.12	**0.05**	8.397	**0.016**		3.403	0.421	6.16	0.081	8.327	**0.017**
Comorbidities	-4.845	0.506	1.21	0.863		-7.648	0.368	8.297	0.242	6.281	0.372
Type of surgery	1.023	0.334	-1.395	0.17		0.037	0.976	0.589	0.568	-1.14	0.265
Leak (complication)	0.108	0.986	4.092	0.495		9.516	0.192	2.872	0.637	14.608	0.116
Approach	-5.621	0.089	-4.689	0.139		-12.112	**0.002**	-3.208	0.319	-4.25	0.183
Stoma (diversion)	16.583	0.051	16.672	**0.041**		-1.733	0.861	11.963	0.149	9.915	0.228
Neoadjuvant therapy	-10.12	0.303	1.898	0.84		-2.825	0.805	-17.053	0.074	-6.741	0.478
Adjuvant therapy	-1.09	0.878	-10.904	0.111		4.435	0.593	-2.096	0.762	3.471	0.614

QLQ-30: Cancer Quality of Life Questionnaire-30

QOL: Quality of life

CI: Confidence interval

**Table 4:** Predictors in QLQ-C29 (*N* = 104)

**Parameter estimates**	**Body image**		**Anxiety**			**Weight**			**Sexual interest (men)**		**Urinary frequency**	
	**B (95% CI)**	***P*** ** value**	**B (95% CI)**		***P*** ** value**	**B (95% CI)**		***P*** ** value**	**B (95% CI)**	***P*** ** value**	**B (95% CI)**	***P*** ** value**
Age	-0.014	0.072	0.039		0.267	0.482		0.138	0.881	**0.001**	0.014	0.048
Sex	-0.33	0.087	1.084		0.213	15.516		0.053	-81.428	**0.001**	-0.305	0.092
Tumor site	0.213	0.311	-0.536		0.573	-7.337		0.403	9.553	0.115	0.135	0.494
Stage	0.201	**0.037**	-0.786		0.071	-12.246		**0.002**	7.757	**0.005**	0.074	0.412
Comorbidities	0.262	0.18	-0.561		0.524	-7.838		0.335	11.803	**0.035**	-0.007	0.971
Type of surgery	-0.015	0.606	-0.12		0.349	0.909		0.442	-0.544	0.505	-0.006	0.81
Leak (complication)	0.134	0.44	-0.722		0.356	-5.334		0.459	6.816	0.171	0.256	0.116
Approach	-0.003	0.977	0.667		0.096	7.347		**0.047**	1.932	0.449	-0.203	**0.015**
Stoma (diversion)	0.329	0.151	-0.144		0.889	-6.039		0.526	-1.061	0.872	0.244	0.255
Neoadjuvant therapy	-0.204	0.441	0.337		0.778	14.927		0.175	-0.001	1	-0.323	0.194
Adjuvant therapy	-0.131	0.451	0.897		0.253	9.682		0.181	-3.415	0.494	0.146	0.37
**Parameter estimates**	**Blood and mucus in stool**		**Urinary incontinence**			**Dysuria**			**Abdominal pain**		**Buttock pain**	
	**B (95% CI)**	***P*** **value**	**B (95% CI)**		***P*** **value**	**B (95% CI)**		***P*** **value**	**B (95% CI)**	***P*** **value**	**B (95% CI)**	***P*** **value**
Age	0.177	0.475	0.295		0.256	0.23		0.318	0.104	0.721	-0.134	0.615
Sex	-6.263	0.306	-5.153		0.422	0.501		0.93	-4.178	0.561	-8.044	0.221
Tumor site	8.244	0.218	1.615		0.818	9.68		0.119	-0.406	0.959	22.803	**0.002**
Stage	1.673	0.584	-0.13		0.968	6.174		**0.03**	8.96	**0.013**	1.669	0.612
Comorbidities	-3.669	0.554	-1.647		0.8	2.284		0.691	-2.495	0.732	-2.875	0.666
Type of surgery	0.541	0.548	0.04		0.967	-0.832		0.321	0.94	0.375	0.528	0.586
Leak (complication)	7.432	0.176	8.501		0.14	5.688		0.265	9.234	0.153	9.08	0.124
Approach	-3.865	0.17	-0.459		0.877	-2.43		0.353	-5.674	0.086	-1.908	0.529
Stoma (diversion)	2.867	0.693	7.666		0.314	-3.024		0.654	16.008	0.06	4.544	0.56
Neoadjuvant therapy	-3.364	0.689	4.248		0.63	-2.354		0.763	-14.272	0.148	2.846	0.753
Adjuvant therapy	-3.371	0.541	11.158		0.054	-7.614		0.137	15.41	**0.017**	1.18	0.842
**Parameter estimates**	**Bloating**		**Dry mouth**			**Hair loss**			**Taste**		**Fecal incontinence**	
	**B (95% CI)**	***P*** **value**	**B (95% CI)**	***P*** **value**		**B (95% CI)**	***P*** **value**		**B (95% CI)**	***P*** **value**	**B (95% CI)**	***P*** **value**
Age	-0.148	0.62	0.133	0.603		-0.419	0.117		-0.32	0.136	-0.45	0.575
Sex	-17.996	**0.015**	-4.547	0.47		-24.753	**0.001**		-9.939	0.061	9.842	0.62
Tumor site	-1.691	0.834	-6.087	0.377		-2.719	0.706		1.87	0.747	28.856	0.183
Stage	2.417	0.513	4.452	0.157		7.378	**0.025**		10.172	**0.001**	-4.278	0.666
Comorbidities	-6.16	0.41	-3.064	0.631		9.569	0.151		2.468	0.646	19.006	0.344
Type of surgery	-1.022	0.348	1.553	0.094		1.007	0.299		0.131	0.867	3.965	0.175
Leak (complication)	11.657	0.079	8.142	0.15		-5.128	0.386		-6.563	0.168	-4.777	0.789
Approach	-5.33	0.117	-4.703	0.105		0.288	0.924		-4.47	0.067	21.824	**0.017**
Stoma (diversion)	19.352	**0.027**	15.223	**0.042**		-7.717	0.323		2.287	0.716	-45.491	0.053
Neoadjuvant therapy	-4.886	0.63	-3.341	0.699		4.655	0.607		-12.652	0.082	27.371	0.315
Adjuvant therapy	-4.227	0.526	4.484	0.43		3.163	0.594		-5.847	0.221	10.199	0.568
**Parameters estimates**		**Sore skin**				**Impotence**				**Dyspareunia**		
		**B (95% CI)**		***P*** **value**		**B (95% CI)**			***P*** **value**	**B (95% CI)**		***P*** **value**
Age		-0.903		0.231		-0.292			0.276	-0.193		0.28
Sex		4.812		0.796		67.441			**0.001**	-44.868		**0.001**
Tumor site		40.862		**0.044**		11.388			0.115	8.298		0.085
Stage		-2.386		0.797		-6.954			**0.035**	4.237		0.054
Comorbidities		19.632		0.297		19.069			**0.004**	3.341		0.454
Type of surgery		4.147		0.13		0.998			0.306	0.574		0.377
Leak (complication)		3.173		0.849		-5.374			0.366	-6.963		0.079
Approach		19.567		**0.022**		3.69			0.226	1.797		0.376
Stoma (diversion)		69.1		**0.002**		-14.785			0.059	1.347		0.797
Neoadjuvant therapy		25.153		0.324		18.539			**0.041**	-10.484		0.083
Adjuvant therapy		7.803		0.642		18.92			**0.002**	-0.63		0.874

## Discussion

Our cohort was characterized by four attributes. First, the participants were relatively young, with an average age of 57 years, and 29% were aged < 50 years, whereas the median age of CRC occurrence in Saudi is 65 years [1]. Second, rectal cancer represented 42.3% (44/104) of the cases, with a ratio of rectal to colon cancer of 1:1.3 compared with a 4:1 ratio reported by a national study [7–11]. Third, 63% of the participants had locally advanced or metastatic cancer, which is in line with the national statistics from 2020 [1]. Finally, approximately 40% of the participants underwent chemotherapy and stoma surgery. All these factors may have a detrimental effect on patient QOL. Nevertheless, 72.4% of patients exhibited a high degree of global health status (score ≥ 66.7). These results are higher than the national scores of 49% [8], 36% [10], and 54% [11]. Several factors may have contributed to this, including longitudinal data after one year of treatment completion, recent advancements in CRC management, high rates of minimally invasive surgery, high rates of sphincter preservation surgery, early stoma closure within 6–8 weeks, a low leakage rate of 4.8%, and the availability of all management modalities in a single center, including chemoradiotherapy, targeted therapy, positron emission tomography scans, and tumor board discussion. Another contributing factor may be the presence of private insurance, which provides patients with a sense of security [14]. Patient dissatisfaction with the health care system is a contributing factor to poor QOL [15]. The global QOL in this cohort aligned with international data [16].

The study group exhibited impairments in functionality, with physical function having the lowest mean at 58. Several researchers have reported similar findings [17]. CRC survivors who received a diagnosis within the past year, had advanced disease, and underwent cancer treatment during the previous month had a higher incidence of physical disability, with rates of 33%, 48%, and 61%, respectively [18]. Patients who received a CRC diagnosis more than 5 years ago, had localized cancer, and had not received recent treatment demonstrated physical functioning levels comparable to those of the general population without cancer [18]. Psychological distress, related to functional disability, was prevalent among CRC survivors with advanced disease or those who had recently undergone cancer treatment, with rates of 46% and 33%, respectively [18]. Nevertheless, this patient group demonstrated the highest mean and best functioning for anxiety on the QLQ-CR29 functional scale, similar to the findings of other local studies [8, 11]. This study revealed that insomnia and fatigue were the most problematic symptoms, in line with previous reports [8, 10, 11]. Several authors have reported a significant correlation between decreased sleep quality and fatigue lasting from 6 weeks to 24 months following treatment of CRC survivors [19–22].

The average score for male libido and body image issues exceeded 66.6. This can be attributed to the higher prevalence of minimally invasive surgery (59.7%) and the avoidance of stoma (60.6%). Another plausible explanation is the relatively young age of the participants, given that their mean age was 57 years. Most studies examining sexual dysfunction following CRC surgery suffer from methodological inaccuracies, preventing them from reaching definitive conclusions. Specifically, these studies are affected by issues such as the definition of sexual satisfaction, the nature of relationships and intimacy between couples, the scale used to assess sexual dysfunction, and the distinction between tumor locations [23]. Sexual dysfunction affects up to 50% of men and 58% of women [24].

The stoma embarrassment score was higher on the symptom scale in our cohort. Stoma presence is associated with feelings of distress, difficulties in stoma care, discomfort, and skin irritation surrounding the stoma. Depression, anxiety, body image issues, and sexual difficulties are more strongly correlated with a permanent stoma than with a temporary stoma [25–27].

This study investigated 11 predictors of the QLQ-30 and QLQ-CR29 scores. Patient age had no effect on functional or symptomatic outcomes in this cohort. This can be attributed to the relatively young age range of individuals in this cohort; nevertheless, male patients with early-onset CRC may have worse QOL than older or female patients do [5, 28]. Among the geriatric population, age is merely a numerical value. A direct relationship exists between QOL and different factors, such as the level of physical activity, absence of sensory deficits, mental well-being, ability to cope with challenges, and degree of independence [29].

Sex disparity is a determinant that impacts QOL owing to differences between men and women in specific domains, such as cognitive function, which is affected more in women [30]. Some women suffer more from fatigue, pain, and physical and social functioning [31].

In accordance with our cohort, a review of 1,021 patients revealed that tumor location did not affect patient health QOL [32]. However, other mentioned effects, especially in rectal cancer, are more pronounced with short-course radiotherapy than with long-course radiotherapy [26]. Patients with rectal cancer in this study underwent short-course radiation as an integral component of their neoadjuvant protocol.

In this cohort, 24% (25/104) reported fecal incontinence as their worst symptom. The location of the anastomosis contributes to the poor defecation function observed in coloanal anastomosis [27]. The overall QOL did not seem to differ among patients who underwent APR with permanent colostomy, colorectal anastomosis, or coloanal anastomosis [27].

Neither the surgical type and approach nor postoperative complications such as leaks affected patient QOL in this study. Potential explanations for this could be the relatively small percentage of leaks in this group, which was 5%, and the patients’ primary focus on achieving a cure rather than choosing a specific surgical method. A previous study reported that laparoscopic surgery has a favorable QOL outcome [33].

In accordance with other studies [14], tumor stage was the only variable that had a significant negative impact on overall health-related QOL and functional and symptom scale scores. Early-stage CRC has better outcomes than locally advanced or metastatic cancer [33]. Establishing systemic treatments, such as targeted therapies and chemotherapy combinations, has a significant effect on improving survival rates and QOL in patients with stage IV CRC [34]. Table 5 provides a comparative summary of studies on the factors influencing quality of life among colorectal cancer patients. Using Ashing-Giwa’s [35] Contextual Model as a guide, our findings show that disease and treatment factors—particularly tumor stage and the presence of a stoma—have the strongest and most consistent influence on patients’ overall well-being and their ability to function socially and in daily roles. Individual factors such as age, sex, and other health conditions affect specific areas, such as physical functioning and sexual health. At the system level, features of our health care setting—centralized cancer services, high use of minimally invasive surgery, and multidisciplinary team care—may help explain why many patients still report good overall quality of life, even with a high proportion of advanced disease. Looking through this contextual lens helps us understand why certain effects appear in specific domains and highlights the need to explore additional influences—such as social support and financial stress—in future studies.

**Table 5:** Comparative summary of studies on factors influencing quality of life in colorectal cancer patients

Study (year)	Setting	*N*	Time since treatment	Key predictors of poorer HRQOL	Most affected domains/symptoms
Present study	Single-center, cross-sectional	104	≥ 12 months	Higher tumor stage; stoma presence and neoadjuvant chemoradiation	Lower physical/role/social; insomnia, fatigue; stoma embarrassment and bloating
Chambers et al. 2012 [14]	Prospective, 5-year	763	Longitudinal (over time)	later stage, permanent stoma, rectal cancer, smoking, being single, low social support and low optimism	Physical function and distress
Dunn et al. 2013 [16]	Prospective	1966	Longitudinal (over time)	Lower optimism, poorer social support, more negative cognitive appraisal, and younger age	Life satisfaction
Momeni et al. 2014 [35]	Single-center, cross-sectional	110	> 12 months	Age < 60; female sex; no insurance; co-morbidity and low income	Emotional well-being, vitality and social function
Almutairi et al. 2016 [8]	Multi-center, cross-sectional	106	different time of survivorship	Advanced disease, recent treatment and insurance	Lower physical functioning and fatigue
Alabbas et al. 2016 [9]	Single center	40	different time of survivorship	Male gender	Pain, appetite loss, constipation, Symptom burden (GI) and complications
Trenti et al. 2018 [27]	Cross-sectional, rectal	358	Post-surgery	Low anastomosis	Bowel function and QoL decrements
Downing et al. 2019 [26]	Population-level, rectal cancer	6713	Longitudinal (over time)	Short-course RT, rectal-specific and stoma	Bowel function and sexual function
Younis et al. 2019 [36]	Multi-center, cross-sectional	40	different time of survivorship	Education level	fatigue, pain, and insomnia
Zhang et al. 2020 [18]	Population cohort	2395	different time of survivorship	Metastatic tumor and recent therapy	Physical function and distress
Abu-Helalah et al. 2022 [10]	Multi-center	115	different time of survivorship	Psychological distress and disease status	Global health and emotional
Qedair et al. 2022 [11]	Single center	118	different time of survivorship	Income and age	Body image and bowel dysfunction

This cross-sectional study has several limitations, with the most significant being the relatively small sample size, which may limit the generalizability of the findings. A smaller sample size can also reduce the statistical power of the study. Other limitations include potential selection bias, as the participants who volunteered may not fully represent the diversity of CRC survivors, and recall bias, as the data relied on self-reported measures. Despite these limitations, this study highlights the need for long-term data and overlooked predictors that influence the QOL of CRC survivors, contributing valuable insights to the field.

Two prominent disease characteristics, tumor stage and stoma presence, significantly diminished global QOL. Stage 4 CRC resulted in a 20-fold reduction in the global health score. Stoma presence resulted in emotional discomfort and a 22-fold decrease in social function scores. The identification of such predictors can allow for close monitoring of patients, helping to minimize complications and enhance outcomes. We propose the establishment of lifestyle offices and two support programs for those with stomas and those with advanced tumors to improve patients’ QOL. This study may help identify additional predictors, optimize resource allocation for patients with critical needs, and foster innovation in management.

## Supplementary Information

Below is the link to the electronic supplementary material.

Supplementary Material 1

## Data Availability

The data included in this research are available in the tables within the manuscript. For further inquiries or access to the data, please contact the corresponding author.
