# Supplementary material for: Predictors of impaired quality of life among colorectal cancer survivors: a cross-sectional study
Source: J Patient Rep Outcomes. 2025 Nov 25;9:136. doi: 10.1186/s41687-025-00971-5 (PMC12647477; doi:10.1186/s41687-025-00971-5)
Supplement: Supplementary file 1 — Supplementary Material 1 [file 41687_2025_971_MOESM1_ESM.docx]

**Supplementary Text**

**Details about EORTC C30 and C29**

The QLCQ-C30 is a comprehensive assessment tool for measuring global health and functional status. It includes a single scale for measuring QOL and five subscales for measuring various aspects of functional status, including physical, role, emotional, cognitive, and social functions. The following 10 components are used to measure CRC symptoms: exhaustion, nausea and vomiting, pain, dyspnea, insomnia, lack of appetite, constipation, diarrhea, and financial difficulties.

The QLQ-CR29 is a functional and symptoms scale that includes questions on body image, anxiety, weight, and sexual interest, among others. It includes a long list of symptoms, such as dysuria, frequent urination, abdominal pain, buttock pain, dry mouth, hair loss, loss of taste, flatulence, fecal incontinence, sore skin, stoma care complications, impotence, and dyspareunia.

The QLQ-30 and QLQ-CR-29 utilize a Likert scale ranging from 1–4, where 1 represents "not at all," 2 "a little," 3 "quite a bit," and 4 "very much." We evaluated the final two items in QLQ-30 using a Likert scale ranging from 1–7, where score of 1 signifies dissatisfaction and 7 represents strong satisfaction. We subjected the Likert scales to the EORTC QLQ-30 scoring manual, initially measuring the raw score and then linearly transforming it into a score range from 0–100. A higher scale score indicates a higher response level. Therefore, a high score on a functional scale indicates a proficient level of functioning, whereas a high score on the global health status or QOL indicates a high QOL. However, a high symptom scale or item score signifies a noteworthy degree of symptomatology or problems.
